# Could sexual selection be driven by the mistaken inferences of young females?

**DOI:** 10.1371/journal.pbio.3002321

**Published:** 2023-10-04

**Authors:** Tamra C. Mendelson, Gail L. Patricelli, Eileen A. Hebets

**Affiliations:** 1 Department of Biological Sciences, University of Maryland, Baltimore County, Baltimore, Maryland, United States of America; 2 Department of Evolution and Ecology, University of California, Davis, California, United States of America; 3 School of Biological Sciences, University of Nebraska, Lincoln, Nebraska, United States of America

## Abstract

This Primer explores a new evolutionary model of mate choice copying, published in PLOS Biology, which aims to reconcile mismatches between theory and data by proposing that juvenile females mistakenly imprint on male phenotypes that were not in fact preferred by the female they copied.

## Teaser

A new evolutionary model of mate choice copying, published in *PLOS Biology*, aims to reconcile mismatches between theory and data by proposing that juvenile females mistakenly imprint on male phenotypes that were not in fact preferred by the female they copied.

The evolutionary origin of female preference is the subject of intense research in behavioral ecology, motivated by Darwin’s once unpopular opinion that the female brain is a powerful agent of selection [1]. Many now hypothesize that a female’s preference confers a fitness advantage. For example, preference for a large nuptial gift would provide direct benefits in the form of nutrition, or a female could receive indirect benefits for her preference if her sons or daughters inherit traits from their father that are adaptive, or just sexy [2]. Preferences can also arise in contexts other than mating. Brains and their sensory systems must adapt to a multitude of life tasks, and those adaptations can generate sensory or perceptual biases that affect which displays will catch a female’s attention or entice her to mate [3]. Females can also learn their preferences by copying the choices of other females [4], by imprinting on adult phenotypes [5], or even by looking at themselves [6].

In all these cases, we are driven to understand the origin of mate choice because we are driven to understand animal ornamentation, and it seems clear in many cases that the opposite sex has something to do with it. But how? Any one or a combination of these mechanisms could explain the evolution of elaborate sexual displays and corresponding preferences in any given animal, and our job as scientists is to figure out which ones, if any, and why. But our job is also to generate new hypotheses, if available options fall short in explaining what we see. This is what DuVal and colleagues offer in this issue of *PLOS Biology* [7], based on years of collective experience observing and modeling the behavior of wild animals.

Their idea is deceptively simple. If a chooser, say, a female, observes another female mating, the observing female is predicted to develop a template—a neural representation of an “attractive mate”—based on the trait that best distinguishes the chosen male from the other males she has observed in the population. If we assume that uncommon traits will be more distinctive, the copying female will imprint on the rarest trait exhibited by the chosen male. For example, if a juvenile observes an adult mating with a male that has red plumage and a long tail, the juvenile will imprint on whichever trait is rarer in the population—the red plumage or the longer tail—regardless of which trait drove the adult female’s choice. If the rare trait was not in fact the choosing female’s target, the young female may start a new fashion trend. The conceptual model thus predicts fluctuations in the target of mate choice across generations, leading to variation in both preference and ornament frequencies over time.

The authors next set their ideas to math. Sexual ornaments are modeled as 2 independent characters (e.g., plumage color and tail length), each with 2 character states (e.g., red versus pink and long versus short). Juvenile females in the model each observe a single mating event by an experienced adult female. The result, depending on the strength of female preference and the strength of viability selection on the ornaments, is that the predicted population fluctuations appear. Variation among individuals in both preferences and traits is maintained in the population, with oscillations between alternative states over time. Notably, a supplementary model provided by the authors, of female preference for rare males alone with no mate choice copying, found similarly extreme oscillations—but only in large groups. Combining mate choice copying and rare male preference thus expanded the conditions under which extreme oscillations in preference and traits could occur.

Like all models, the inferred attractiveness model makes simplifying assumptions that do not fully capture the complexity of nature, but rather allow us to mathematically explore an evolutionary process in a simpler form. However, the model also assumes complex cognitive mechanisms of female preference. The applicability of the model to any given system hangs on empirical support for these mechanisms, so it’s worth exploring what support currently exists and what remains to be tested.

One key cognitive mechanism of inferred attractiveness is the social learning of mates; juvenile females observe adults mating and imprint on the traits of the male. This type of mate choice learning, referred to as mate choice copying or imprinting, has been explored empirically and theoretically (reviewed in [8]), and it has been demonstrated in the laboratory for a variety of organisms, including insects and arachnids (references in [7,9]). The extent to which this process occurs in nature is unknown for most taxa, however, as are the physiological or cognitive mechanisms underlying it, notably the relative impact of social versus nonsocial (personal) information in generating a mate choice. We do know that it should depend on a number of factors that affect the opportunity and capacity for mate choice learning (e.g., overlapping generations, density, encounter rates, copulation duration, habitat complexity, sensory modality, and memory capacity). Provided favorable conditions are met, in species where copying occurs, we indeed have reason to expect that it will influence preferences and thus selection on display traits.

Another key cognitive mechanism is that juvenile copiers learn or imprint on the rarest trait of the male they observed mating. They remember this trait until they are adults, at which time they choose a male with that same rare trait variant. This mechanism makes some testable assumptions. First, it assumes that females pay selective attention to one discrete trait in forming a category or template for “attractive male,” rather than paying distributed attention to multiple traits. If indeed females are forming categories, the evidence for this assumption is mixed. For example, in some categorization tasks, adult humans do focus on single traits that best predict a category; however, some studies show that children and pigeons tend instead to show distributed attention [10]. An exploration of the psychological concepts of “information integration” (integrating multiple stimulus dimensions) versus “rule based” (single stimulus dimension) category learning seems especially relevant here [11]. In other words, it remains to be tested whether a juvenile female observing the mating of a male with red feathers and a long tail learns only one of those traits or whether she learns both red plumage and long tail. If she learns both, and the combination is novel, then over time, novel traits could be added to the template, which might help to explain why highly polygynous species often have many ornaments.

The second testable assumption is that juveniles form a category or template based on the most rare trait variant of the chosen male—even if the rarer trait is only marginally more rare—and then form a preference for males with that variant. Empirical examples of such a mechanism were not explicitly provided, but experiments in fruit flies that allowed females to observe multiple mating pairs found instead a conformist bias, with females imprinting on the trait most common among mating males [12]. If such conformist cognitive biases are widespread, they may counteract the process modelled here, especially if juveniles can observe multiple matings.

In addition to exploring these cognitive mechanisms, the model gives theoreticians and empiricists plenty to do. Extensions of the model that explicitly account for sensory or perceptual biases could be instructive, as would models that include observations of multiple matings, or traits that vary continuously rather than categorically. Empiricists might ask how often the oscillations in frequency of display traits and preferences generated by the model occur in nature. Data from a long-term study of sexual selection in the wild, like DuVal’s study of lance-tailed manakins, may be ideal to test this.

Ultimately, the inferred attractiveness model is a thought-provoking way of modeling mate choice, and it suggests that mate choice copying and template learning might be an underrated source of variation in preferences and display traits within and among populations. The model also provides a possible new solution to the lek paradox, explaining how genetic variation in traits and preferences can be maintained in polygynous mating systems. Multiple open and interesting questions are posed by its key assumptions and predicted outcomes, and we hope that the model inspires further research to address them. We are excited to see the results.
